# Dimethyl 4-(4-ethoxy­phen­yl)-2,6-dimethyl-1,4-dihydro­pyridine-3,5-dicarboxyl­ate

**DOI:** 10.1107/S1600536809033364

**Published:** 2009-08-26

**Authors:** Hoong-Kun Fun, Ching Kheng Quah, B. Palakshi Reddy, S. Sarveswari, V. Vijayakumar

**Affiliations:** a X-ray Crystallography Unit, School of Physics, Universiti Sains Malaysia, 11800 USM, Penang, Malaysia; b Organic Chemistry Division, School of Science and Humanities, VIT University, Vellore 632 014, India

## Abstract

In the title mol­ecule, C_19_H_23_NO_5_, the dihedral angle formed by the benzene ring and the planar part of the dihydro­pyridine ring is 83.52 (5)°. The dihydro­pyridine ring adopts a flattened boat conformation. In the crystal, mol­ecules are linked by N—H⋯O hydrogen bonds, generating chains running parallel to [100]. The crystal structure is consolidated by C—H⋯O contacts.

## Related literature

For general background to Hantzsch 1,4-dihydro­pyridines (1,4-DHPS), see: Gaudio *et al.* (1994 ▶); Bocker & Guengerich (1986 ▶); Gordeev *et al.* (1996 ▶); Sunkel *et al.* (1992 ▶); Vo *et al.* (1995 ▶); Cooper *et al.* (1992 ▶). For a related structure, see: Fun *et al.* (2009 ▶). For hydrogen-bond motifs, see: Bernstein *et al.* (1995 ▶). For geometric analysis, see: Cremer & Pople (1975 ▶). For the stability of the temperature controller used for the data collection, see: Cosier & Glazer (1986 ▶).
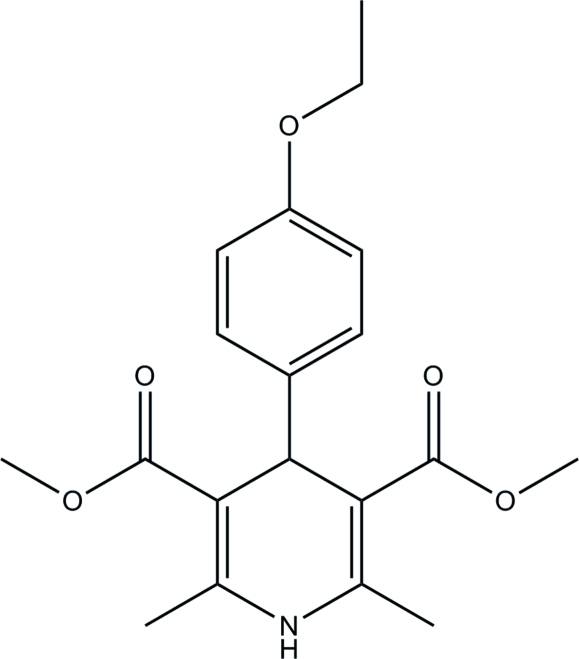


## Experimental

### 

#### Crystal data

C_19_H_23_NO_5_
*M* *_r_* = 345.38
Triclinic, 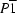

*a* = 7.4108 (1) Å
*b* = 9.7459 (2) Å
*c* = 12.3359 (2) Å
α = 87.412 (1)°
β = 86.244 (1)°
γ = 76.402 (1)°
*V* = 863.72 (3) Å^3^
*Z* = 2
Mo *K*α radiation
μ = 0.10 mm^−1^
*T* = 100 K
0.39 × 0.33 × 0.19 mm

#### Data collection

Bruker SMART APEXII CCD area-detector diffractometer
Absorption correction: multi-scan (**SADABS**; Bruker, 2005 ▶) *T*_min_ = 0.963, *T*_max_ = 0.982
26880 measured reflections
4579 independent reflections
3972 reflections with *I* > 2σ(*I*)
*R*_int_ = 0.026

#### Refinement

*R*[*F* ^2^ > 2σ(*F* ^2^)] = 0.036
*wR*(*F* ^2^) = 0.099
*S* = 1.05
4579 reflections
235 parameters
H atoms treated by a mixture of independent and constrained refinement
Δρ_max_ = 0.38 e Å^−3^
Δρ_min_ = −0.23 e Å^−3^

### 

Data collection: *APEX2* (Bruker, 2005 ▶); cell refinement: *SAINT* (Bruker, 2005 ▶); data reduction: *SAINT*; program(s) used to solve structure: *SHELXTL* (Sheldrick, 2008 ▶); program(s) used to refine structure: *SHELXTL*; molecular graphics: *SHELXTL*; software used to prepare material for publication: *SHELXTL* and *PLATON* (Spek, 2009 ▶).

## Supplementary Material

Crystal structure: contains datablocks global, I. DOI: 10.1107/S1600536809033364/tk2529sup1.cif

Structure factors: contains datablocks I. DOI: 10.1107/S1600536809033364/tk2529Isup2.hkl

Additional supplementary materials:  crystallographic information; 3D view; checkCIF report

## Figures and Tables

**Table 1:** Hydrogen-bond geometry (Å, °)

*D*—H⋯*A*	*D*—H	H⋯*A*	*D*⋯*A*	*D*—H⋯*A*
N1—H1*N*1⋯O4^i^	0.854 (15)	2.230 (15)	3.0710 (11)	168.0 (13)
C4—H4*A*⋯O1^ii^	0.93	2.58	3.5104 (12)	174
C15—H15*A*⋯O1^iii^	0.96	2.60	3.5500 (14)	172
C19—H19*B*⋯O4^i^	0.96	2.57	3.4677 (12)	155

Symmetry codes: (i) 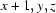; (ii) 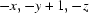; (iii) 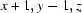.

**Fig. 1.**
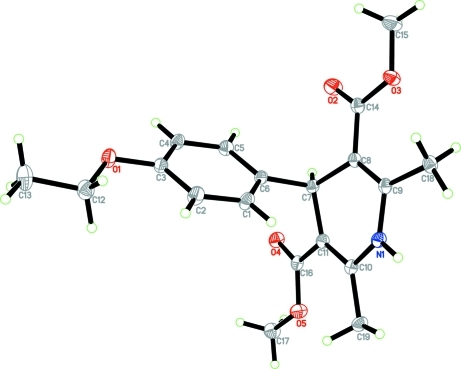
The molecular structure of (I), showing 50% probability displacement ellipsoids and the atom-numbering scheme.

**Fig. 2.**
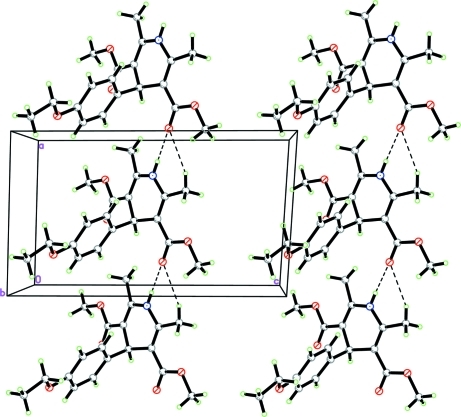
The crystal packing of (I), viewed along the b axis, showing the R21(6) ring motifs. The dashed lines indicate N-H···O hydrogen bonds and C-H···O contacts.

## supplementary crystallographic information

### Comment

Hantzsch 1,4-dihydropyridines (1,4-DHPS) are biologically active compounds which include various vasodilator, anti-hypertensive, bronchodilator, hepatoprotective, anti-tumor, anti-mutagenic, geroprotective, and anti-diabetic agents (Gaudio *et al.*, 1994). Nifedipine, Nitrendipine and Nimodipine have found commercial utility as calcium channel blockers (Bocker & Guengerich, 1986; Gordeev *et al.*, 1996). For the treatment of congestive heart failure, a number of DHP calcium antagonists have been introduced (Sunkel *et al.*, 1992; Vo *et al.*, 1995). Some DHPs have been introduced as neuroprotectants and cognition enhancers. In addition, a number of DHPs with platelet anti-aggregatory activity have also been discovered (Cooper *et al.*, 1992).

The geometric parameters in (I), Fig. 1, are comparable to those in a closely related structure (Fun *et al.*, 2009). The benzene ring (C1—C6) and dihydropyridine ring (C7—C11/N1) are nearly perpendicular as seen in the angle of 83.52 (5)° between their least-squares planes. The dihydropyridine ring adopts a flattened boat conformation with puckering parameters (Cremer & Pople, 1975) Q = 0.2688 (10) Å; Θ = 73.7 (2)° and φ = 183.6 (2)°, with atoms N1 and C7 deviating by 0.125 (1) and 0.172 (1) Å, respectively, from the mean plane of the dihydropyridine ring.

In the solid-state (Fig. 2), the molecules are linked *via* N1—H1N1···O4 hydrogen bonds (Table 1) to generate supramolecular chains running parallel to the [1 0 0] direction. The O4 atom also participates in a C19—H19B···O4 contact to generate, along with the N1—H1N1···O4 hydrogen bond, a *R*_2_^1^(6) ring motif (Bernstein *et al.*, 1995); Fig. 2. Molecules are further consolidated by intermolecular C—H···O interactions (Table 1).

### Experimental

Compound (I) was prepared according to the Hantzsch pyridine synthesis. A mixture of 4-ethoxybenzaldehyde (10 mmol), methylacetoacetate (20 mmol) and ammonium acetate (10 mmol) were heated at 353 K for 3 h (monitored by TLC). After completion of the reaction, the mixture was cooled to room temperature and kept for 2 days to get the solid product. The solid was extracted using diethyl ether and the mother liquors kept for crystallization. The purity of the crude product was checked through TLC and recrystallized using acetone and ether; *M.p.* 403–405 K. IR (KBr): υ: 3361, 2985, 2948, 1682, 1652, 1485, 1235 cm^-1^.

### Refinement

The N-bound H atom was located from a difference Fourier map and refined freely. The other H atoms were placed in calculated positions with C–H = 0.93 – 0.98 Å, and refined using a riding model with *U*_iso_(H) = 1.2 or 1.5 *U*_eq_(C). A rotating-group model was applied for the methyl groups.

### Crystal data

**Table:**

C_19_H_23_NO_5_	*Z* = 2
*M**_r_* = 345.38	*F*(000) = 368
Triclinic, *P*1	*D*_x_ = 1.328 Mg m^−^^3^
Hall symbol: -P 1	Mo *K*α radiation, λ = 0.71073 Å
*a* = 7.4108 (1) Å	Cell parameters from 9914 reflections
*b* = 9.7459 (2) Å	θ = 2.7–37.4°
*c* = 12.3359 (2) Å	µ = 0.10 mm^−^^1^
α = 87.412 (1)°	*T* = 100 K
β = 86.244 (1)°	Block, colourless
γ = 76.402 (1)°	0.39 × 0.33 × 0.19 mm
*V* = 863.72 (3) Å^3^	

### Data collection

**Table:**

Bruker SMART APEXII CCD area-detector diffractometer	4579 independent reflections
Radiation source: fine-focus sealed tube	3972 reflections with *I* > 2σ(*I*)
graphite	*R*_int_ = 0.026
φ and ω scans	θ_max_ = 29.0°, θ_min_ = 2.2°
Absorption correction: multi-scan (*SADABS*; Bruker, 2005)	*h* = −10→9
*T*_min_ = 0.963, *T*_max_ = 0.982	*k* = −13→13
26880 measured reflections	*l* = −16→16

### Refinement

**Table:**

Refinement on *F*^2^	Primary atom site location: structure-invariant direct methods
Least-squares matrix: full	Secondary atom site location: difference Fourier map
*R*[*F*^2^ > 2σ(*F*^2^)] = 0.036	Hydrogen site location: inferred from neighbouring sites
*wR*(*F*^2^) = 0.099	H atoms treated by a mixture of independent and constrained refinement
*S* = 1.05	*w* = 1/[σ^2^(*F*_o_^2^) + (0.0475*P*)^2^ + 0.2882*P*] where *P* = (*F*_o_^2^ + 2*F*_c_^2^)/3
4579 reflections	(Δ/σ)_max_ = 0.001
235 parameters	Δρ_max_ = 0.38 e Å^−^^3^
0 restraints	Δρ_min_ = −0.23 e Å^−^^3^

### Special details

**Table:**

Experimental. The crystal was placed in the cold stream of an Oxford Cyrosystems Cobra open-flow nitrogen cryostat (Cosier & Glazer, 1986) operating at 100.0 (1) K.
Geometry. All e.s.d.'s (except the e.s.d. in the dihedral angle between two l.s. planes) are estimated using the full covariance matrix. The cell e.s.d.'s are taken into account individually in the estimation of e.s.d.'s in distances, angles and torsion angles; correlations between e.s.d.'s in cell parameters are only used when they are defined by crystal symmetry. An approximate (isotropic) treatment of cell e.s.d.'s is used for estimating e.s.d.'s involving l.s. planes.
Refinement. Refinement of *F*^2^ against ALL reflections. The weighted *R*-factor *wR* and goodness of fit *S* are based on *F*^2^, conventional *R*-factors *R* are based on *F*, with *F* set to zero for negative *F*^2^. The threshold expression of *F*^2^ > σ(*F*^2^) is used only for calculating *R*-factors(gt) *etc*. and is not relevant to the choice of reflections for refinement. *R*-factors based on *F*^2^ are statistically about twice as large as those based on *F*, and *R*- factors based on ALL data will be even larger.

### Fractional atomic coordinates and isotropic or equivalent isotropic displacement parameters (Å^2^)

**Table:**

	*x*	*y*	*z*	*U*_iso_*/*U*_eq_	
O1	0.19861 (10)	0.60173 (8)	0.03818 (6)	0.01954 (16)	
O2	0.44712 (10)	−0.06125 (8)	0.22047 (7)	0.02351 (17)	
O3	0.75904 (10)	−0.12488 (7)	0.20146 (6)	0.01800 (15)	
O4	0.12920 (9)	0.26642 (8)	0.49547 (6)	0.01857 (16)	
O5	0.28689 (10)	0.36872 (8)	0.60451 (6)	0.01980 (16)	
N1	0.78173 (11)	0.17632 (9)	0.43091 (7)	0.01522 (17)	
C1	0.47641 (14)	0.38112 (11)	0.24725 (8)	0.0186 (2)	
H1A	0.5811	0.3780	0.2858	0.022*	
C2	0.42251 (14)	0.49057 (11)	0.17125 (9)	0.0205 (2)	
H2A	0.4905	0.5593	0.1594	0.025*	
C3	0.26590 (13)	0.49643 (10)	0.11306 (8)	0.01547 (19)	
C4	0.16801 (13)	0.39106 (10)	0.12993 (8)	0.01699 (19)	
H4A	0.0646	0.3934	0.0904	0.020*	
C5	0.22500 (13)	0.28219 (10)	0.20605 (8)	0.01653 (19)	
H5A	0.1593	0.2118	0.2163	0.020*	
C6	0.37885 (12)	0.27631 (10)	0.26738 (7)	0.01368 (18)	
C7	0.43800 (12)	0.16185 (10)	0.35541 (7)	0.01319 (17)	
H7A	0.3410	0.1087	0.3666	0.016*	
C8	0.61931 (12)	0.05947 (10)	0.32117 (7)	0.01377 (18)	
C9	0.78381 (13)	0.07461 (10)	0.35543 (8)	0.01377 (18)	
C10	0.62373 (13)	0.24334 (10)	0.49089 (8)	0.01427 (18)	
C11	0.45520 (12)	0.22955 (10)	0.46159 (7)	0.01390 (18)	
C12	0.28242 (15)	0.72105 (11)	0.03018 (9)	0.0222 (2)	
H12A	0.4125	0.6916	0.0059	0.027*	
H12B	0.2744	0.7636	0.1004	0.027*	
C13	0.17764 (17)	0.82565 (13)	−0.05091 (10)	0.0293 (3)	
H13A	0.2306	0.9067	−0.0585	0.044*	
H13B	0.0494	0.8544	−0.0258	0.044*	
H13C	0.1863	0.7823	−0.1200	0.044*	
C14	0.59716 (13)	−0.04608 (10)	0.24493 (8)	0.01548 (18)	
C15	0.73788 (15)	−0.22168 (12)	0.12099 (9)	0.0234 (2)	
H15A	0.8579	−0.2777	0.0980	0.035*	
H15B	0.6800	−0.1695	0.0596	0.035*	
H15C	0.6617	−0.2824	0.1517	0.035*	
C16	0.27731 (13)	0.28733 (10)	0.52057 (8)	0.01461 (18)	
C17	0.11161 (14)	0.43650 (12)	0.65823 (9)	0.0214 (2)	
H17A	0.1336	0.4930	0.7155	0.032*	
H17B	0.0500	0.3659	0.6882	0.032*	
H17C	0.0348	0.4957	0.6067	0.032*	
C18	0.97650 (13)	−0.00902 (10)	0.32325 (8)	0.01687 (19)	
H18A	1.0010	0.0034	0.2465	0.025*	
H18B	0.9854	−0.1073	0.3408	0.025*	
H18C	1.0659	0.0235	0.3619	0.025*	
C19	0.66430 (13)	0.32411 (11)	0.58374 (8)	0.01801 (19)	
H19A	0.5904	0.4192	0.5801	0.027*	
H19B	0.7937	0.3254	0.5792	0.027*	
H19C	0.6347	0.2793	0.6513	0.027*	
H1N1	0.886 (2)	0.1880 (15)	0.4495 (12)	0.027 (3)*	

### Atomic displacement parameters (Å^2^)

**Table:**

	*U*^11^	*U*^22^	*U*^33^	*U*^12^	*U*^13^	*U*^23^
O1	0.0198 (4)	0.0181 (3)	0.0215 (4)	−0.0055 (3)	−0.0063 (3)	0.0054 (3)
O2	0.0157 (3)	0.0248 (4)	0.0319 (4)	−0.0066 (3)	−0.0035 (3)	−0.0084 (3)
O3	0.0157 (3)	0.0167 (3)	0.0220 (4)	−0.0044 (3)	0.0005 (3)	−0.0051 (3)
O4	0.0112 (3)	0.0236 (4)	0.0213 (4)	−0.0047 (3)	−0.0010 (3)	−0.0018 (3)
O5	0.0128 (3)	0.0258 (4)	0.0209 (4)	−0.0040 (3)	0.0011 (3)	−0.0073 (3)
N1	0.0099 (4)	0.0184 (4)	0.0181 (4)	−0.0042 (3)	−0.0022 (3)	−0.0015 (3)
C1	0.0154 (4)	0.0212 (5)	0.0214 (5)	−0.0075 (4)	−0.0074 (4)	0.0029 (4)
C2	0.0202 (5)	0.0200 (5)	0.0244 (5)	−0.0104 (4)	−0.0067 (4)	0.0040 (4)
C3	0.0153 (4)	0.0158 (4)	0.0144 (4)	−0.0017 (3)	−0.0015 (3)	−0.0002 (3)
C4	0.0142 (4)	0.0197 (5)	0.0178 (4)	−0.0043 (3)	−0.0046 (3)	−0.0001 (4)
C5	0.0149 (4)	0.0169 (4)	0.0194 (5)	−0.0066 (3)	−0.0033 (3)	0.0003 (4)
C6	0.0120 (4)	0.0144 (4)	0.0143 (4)	−0.0021 (3)	−0.0012 (3)	−0.0013 (3)
C7	0.0100 (4)	0.0144 (4)	0.0158 (4)	−0.0038 (3)	−0.0021 (3)	0.0000 (3)
C8	0.0120 (4)	0.0135 (4)	0.0159 (4)	−0.0034 (3)	−0.0009 (3)	0.0008 (3)
C9	0.0128 (4)	0.0130 (4)	0.0154 (4)	−0.0033 (3)	−0.0008 (3)	0.0019 (3)
C10	0.0132 (4)	0.0149 (4)	0.0147 (4)	−0.0033 (3)	−0.0016 (3)	0.0013 (3)
C11	0.0115 (4)	0.0152 (4)	0.0151 (4)	−0.0034 (3)	−0.0009 (3)	0.0004 (3)
C12	0.0249 (5)	0.0192 (5)	0.0238 (5)	−0.0078 (4)	−0.0035 (4)	0.0042 (4)
C13	0.0284 (6)	0.0243 (5)	0.0333 (6)	−0.0047 (4)	−0.0020 (5)	0.0120 (5)
C14	0.0149 (4)	0.0138 (4)	0.0178 (4)	−0.0040 (3)	−0.0009 (3)	0.0012 (3)
C15	0.0231 (5)	0.0214 (5)	0.0270 (5)	−0.0065 (4)	0.0003 (4)	−0.0093 (4)
C16	0.0130 (4)	0.0157 (4)	0.0147 (4)	−0.0028 (3)	−0.0011 (3)	0.0023 (3)
C17	0.0153 (5)	0.0247 (5)	0.0229 (5)	−0.0019 (4)	0.0026 (4)	−0.0055 (4)
C18	0.0112 (4)	0.0172 (4)	0.0221 (5)	−0.0029 (3)	−0.0012 (3)	−0.0005 (4)
C19	0.0135 (4)	0.0222 (5)	0.0192 (5)	−0.0050 (3)	−0.0033 (3)	−0.0033 (4)

### Geometric parameters (Å, °)

**Table:**

O1—C3	1.3745 (11)	C8—C9	1.3580 (13)
O1—C12	1.4384 (13)	C8—C14	1.4686 (13)
O2—C14	1.2143 (12)	C9—C18	1.5062 (13)
O3—C14	1.3551 (11)	C10—C11	1.3605 (12)
O3—C15	1.4403 (12)	C10—C19	1.5033 (13)
O4—C16	1.2237 (11)	C11—C16	1.4651 (13)
O5—C16	1.3479 (12)	C12—C13	1.5100 (15)
O5—C17	1.4443 (12)	C12—H12A	0.9700
N1—C10	1.3841 (12)	C12—H12B	0.9700
N1—C9	1.3872 (12)	C13—H13A	0.9600
N1—H1N1	0.852 (15)	C13—H13B	0.9600
C1—C6	1.3888 (13)	C13—H13C	0.9600
C1—C2	1.3909 (14)	C15—H15A	0.9600
C1—H1A	0.9300	C15—H15B	0.9600
C2—C3	1.3928 (13)	C15—H15C	0.9600
C2—H2A	0.9300	C17—H17A	0.9600
C3—C4	1.3912 (14)	C17—H17B	0.9600
C4—C5	1.3919 (13)	C17—H17C	0.9600
C4—H4A	0.9300	C18—H18A	0.9600
C5—C6	1.3978 (12)	C18—H18B	0.9600
C5—H5A	0.9300	C18—H18C	0.9600
C6—C7	1.5293 (12)	C19—H19A	0.9600
C7—C11	1.5188 (13)	C19—H19B	0.9600
C7—C8	1.5207 (12)	C19—H19C	0.9600
C7—H7A	0.9800		
			
C3—O1—C12	117.11 (8)	O1—C12—C13	107.30 (9)
C14—O3—C15	114.71 (8)	O1—C12—H12A	110.3
C16—O5—C17	116.10 (8)	C13—C12—H12A	110.3
C10—N1—C9	123.78 (8)	O1—C12—H12B	110.3
C10—N1—H1N1	117.3 (10)	C13—C12—H12B	110.3
C9—N1—H1N1	118.0 (10)	H12A—C12—H12B	108.5
C6—C1—C2	122.08 (9)	C12—C13—H13A	109.5
C6—C1—H1A	119.0	C12—C13—H13B	109.5
C2—C1—H1A	119.0	H13A—C13—H13B	109.5
C1—C2—C3	119.51 (9)	C12—C13—H13C	109.5
C1—C2—H2A	120.2	H13A—C13—H13C	109.5
C3—C2—H2A	120.2	H13B—C13—H13C	109.5
O1—C3—C4	116.37 (8)	O2—C14—O3	121.92 (9)
O1—C3—C2	124.04 (9)	O2—C14—C8	123.53 (9)
C4—C3—C2	119.58 (9)	O3—C14—C8	114.53 (8)
C3—C4—C5	119.90 (9)	O3—C15—H15A	109.5
C3—C4—H4A	120.1	O3—C15—H15B	109.5
C5—C4—H4A	120.1	H15A—C15—H15B	109.5
C4—C5—C6	121.45 (9)	O3—C15—H15C	109.5
C4—C5—H5A	119.3	H15A—C15—H15C	109.5
C6—C5—H5A	119.3	H15B—C15—H15C	109.5
C1—C6—C5	117.45 (9)	O4—C16—O5	121.62 (9)
C1—C6—C7	120.24 (8)	O4—C16—C11	123.31 (9)
C5—C6—C7	122.30 (8)	O5—C16—C11	115.06 (8)
C11—C7—C8	110.84 (7)	O5—C17—H17A	109.5
C11—C7—C6	109.88 (7)	O5—C17—H17B	109.5
C8—C7—C6	111.28 (7)	H17A—C17—H17B	109.5
C11—C7—H7A	108.2	O5—C17—H17C	109.5
C8—C7—H7A	108.2	H17A—C17—H17C	109.5
C6—C7—H7A	108.2	H17B—C17—H17C	109.5
C9—C8—C14	125.40 (9)	C9—C18—H18A	109.5
C9—C8—C7	120.71 (8)	C9—C18—H18B	109.5
C14—C8—C7	113.77 (8)	H18A—C18—H18B	109.5
C8—C9—N1	118.66 (8)	C9—C18—H18C	109.5
C8—C9—C18	127.97 (9)	H18A—C18—H18C	109.5
N1—C9—C18	113.36 (8)	H18B—C18—H18C	109.5
C11—C10—N1	118.71 (8)	C10—C19—H19A	109.5
C11—C10—C19	127.90 (9)	C10—C19—H19B	109.5
N1—C10—C19	113.39 (8)	H19A—C19—H19B	109.5
C10—C11—C16	124.96 (9)	C10—C19—H19C	109.5
C10—C11—C7	120.47 (8)	H19A—C19—H19C	109.5
C16—C11—C7	114.33 (8)	H19B—C19—H19C	109.5
			
C6—C1—C2—C3	0.06 (16)	C10—N1—C9—C18	−164.68 (8)
C12—O1—C3—C4	171.89 (9)	C9—N1—C10—C11	−12.56 (14)
C12—O1—C3—C2	−7.98 (14)	C9—N1—C10—C19	167.12 (9)
C1—C2—C3—O1	178.41 (9)	N1—C10—C11—C16	176.37 (8)
C1—C2—C3—C4	−1.46 (15)	C19—C10—C11—C16	−3.25 (16)
O1—C3—C4—C5	−178.70 (9)	N1—C10—C11—C7	−9.52 (13)
C2—C3—C4—C5	1.18 (15)	C19—C10—C11—C7	170.86 (9)
C3—C4—C5—C6	0.51 (15)	C8—C7—C11—C10	26.43 (12)
C2—C1—C6—C5	1.58 (15)	C6—C7—C11—C10	−96.98 (10)
C2—C1—C6—C7	−177.17 (9)	C8—C7—C11—C16	−158.87 (8)
C4—C5—C6—C1	−1.86 (15)	C6—C7—C11—C16	77.72 (10)
C4—C5—C6—C7	176.86 (9)	C3—O1—C12—C13	−176.21 (9)
C1—C6—C7—C11	51.88 (11)	C15—O3—C14—O2	2.92 (14)
C5—C6—C7—C11	−126.81 (9)	C15—O3—C14—C8	−175.86 (8)
C1—C6—C7—C8	−71.27 (11)	C9—C8—C14—O2	176.04 (10)
C5—C6—C7—C8	110.05 (10)	C7—C8—C14—O2	−7.83 (14)
C11—C7—C8—C9	−24.86 (12)	C9—C8—C14—O3	−5.20 (14)
C6—C7—C8—C9	97.74 (10)	C7—C8—C14—O3	170.92 (8)
C11—C7—C8—C14	158.81 (8)	C17—O5—C16—O4	−3.79 (13)
C6—C7—C8—C14	−78.58 (10)	C17—O5—C16—C11	175.00 (8)
C14—C8—C9—N1	−177.70 (8)	C10—C11—C16—O4	−175.82 (9)
C7—C8—C9—N1	6.43 (13)	C7—C11—C16—O4	9.75 (13)
C14—C8—C9—C18	0.95 (16)	C10—C11—C16—O5	5.42 (14)
C7—C8—C9—C18	−174.92 (9)	C7—C11—C16—O5	−169.02 (8)
C10—N1—C9—C8	14.15 (14)		

### Hydrogen-bond geometry (Å, °)

**Table:**

*D*—H···*A*	*D*—H	H···*A*	*D*···*A*	*D*—H···*A*
N1—H1N1···O4^i^	0.854 (15)	2.230 (15)	3.0710 (11)	168.0 (13)
C4—H4A···O1^ii^	0.93	2.58	3.5104 (12)	174
C15—H15A···O1^iii^	0.96	2.60	3.5500 (14)	172
C19—H19B···O4^i^	0.96	2.57	3.4677 (12)	155

Symmetry codes: (i) *x*+1, *y*, *z*; (ii) −*x*, −*y*+1, −*z*; (iii) *x*+1, *y*−1, *z*.

## References

[bb1] Bernstein, J., Davis, R. E., Shimoni, L. & Chang, N.-L. (1995). *Angew. Chem. Int. Ed. Engl.* **34**, 1555–1573.

[bb2] Bocker, R. H. & Guengerich, F. P. (1986). *J. Med. Chem.* **28**, 1596–1603. 10.1021/jm00159a007 3746811

[bb3] Bruker (2005). *APEX2*, *SAINT* and *SADABS* Bruker AXS Inc., Madison, Wisconsin, USA.

[bb4] Cooper, K., Fray, M. J., Parry, M. J., Richardson, K. & Steele, J. (1992). *J. Med. Chem.* **35**, 3115–3129. 10.1021/jm00095a005 1507200

[bb5] Cosier, J. & Glazer, A. M. (1986). *J. Appl. Cryst.* **19**, 105–107.

[bb6] Cremer, D. & Pople, J. A. (1975). *J. Am. Chem. Soc.* **97**, 1354–1358.

[bb7] Fun, H.-K., Goh, J. H., Reddy, B. P., Sarveswari, S. & Vijayakumar, V. (2009). *Acta Cryst.* E**65**, o2247–o2248. 10.1107/S160053680903339X PMC2969936 21577645

[bb8] Gaudio, A. C., Korolkovas, A. & Takahata, Y. J. (1994). *Pharm. Sci.* **83**, 1110–1115. 10.1002/jps.2600830809 7983594

[bb9] Gordeev, M. F., Patel, D. V. & Gordon, E. M. (1996). *J. Org. Chem.* **61**, 924–928.

[bb10] Sheldrick, G. M. (2008). *Acta Cryst.* A**64**, 112–122. 10.1107/S0108767307043930 18156677

[bb11] Spek, A. L. (2009). *Acta Cryst.* D**65**, 148–155. 10.1107/S090744490804362X PMC2631630 19171970

[bb12] Sunkel, C. E., Fau de Casa-Juana, M., Santos, L., Garcia, A. G., Artalejo, C. R., Villarroya, M., Gonzalez-Morales, M. A., Lopez, M. G. & Cillero, J. (1992). *J. Med. Chem.* **35**, 2407–2414. 10.1021/jm00091a008 1377748

[bb13] Vo, D., Matowe, W. C., Ramesh, M., Iqbal, N., Wolowyk, M. W., Howlett, S. E. & Knaus, E. E. (1995). *J. Med. Chem.* **38**, 2851–2859. 10.1021/jm00015a007 7543577

